# Association of medial collateral ligament complex injuries with anterior cruciate ligament ruptures based on posterolateral tibial plateau injuries

**DOI:** 10.1186/s40798-023-00611-6

**Published:** 2023-08-08

**Authors:** Fidelius Von Rehlingen-Prinz, Miriam Leiderer, Julius Dehoust, Tobias Dust, Birgitt Kowald, Karl-Heinz Frosch, Kaywan Izadpanah, Frank Oliver Henes, Matthias Krause

**Affiliations:** 1 Department of Trauma and Orthopaedic Surgery, University Medical Center Hamburg-Eppendorf, Martinistraße 52, 20246 Hamburg, Germany; 2 Department of Diagnostic and Interventional Radiology and Nuclear Medicine, University Medical Center Hamburg-Eppendorf, Martinistrasse 52, 20251 Hamburg, Germany; 3 Department of Trauma Surgery, Orthopaedics and Sports Traumatology, BG Hospital Hamburg, Bergedorfer Str. 10, 21033 Hamburg, Germany; 4 Department of Diagnostic and Interventional Radiology, BG Hospital Hamburg, Bergedorfer Str. 10, 21033 Hamburg, Germany; 5 Department of Orthopaedic and Trauma Surgery, University Medical Center Freiburg, Hugstetter Strasse 55, 79106 Freiburg, Germany

**Keywords:** Anteromedial rotatory instability, Medial collateral ligament complex, Deep MCL, Magnetic resonance imaging, Anterior cruciate ligament injury

## Abstract

**Background:**

The combined injury of the medial collateral ligament complex and the anterior cruciate ligament (ACL) is the most common two ligament injury of the knee. Additional injuries to the medial capsuloligamentous structures are associated with rotational instability and a high failure rate of ACL reconstruction. The study aimed to analyze the specific pattern of medial injuries and their associated risk factors, with the goal of enabling early diagnosis and initiating appropriate therapeutic interventions, if necessary.

**Results:**

Between January 2017 and December 2018, 151 patients with acute ACL ruptures with a mean age of 32 ± 12 years were included in this study. The MRIs performed during the acute phase were analyzed by four independent investigators—two radiologists and two orthopedic surgeons. The trauma impact on the posterolateral tibial plateau and associated injuries to the medial complex (POL, dMCL, and sMCL) were examined and revealed an injury to the medial collateral ligament complex in 34.4% of the patients. The dMCL was the most frequently injured structure (92.2%). A dMCL injury was significantly associated with an increase in trauma severity at the posterolateral tibial plateau (*p* < 0.02) and additional injuries to the sMCL (OR 4.702, 95% CL 1.3–133.3, *p* = 0.03) and POL (OR 20.818, 95% CL 5.9–84.4, *p* < 0.0001). Isolated injuries to the sMCL were not observed. Significant risk factors for acquiring an sMCL injury were age (*p* < 0.01) and injury to the lateral meniscus (*p* < 0.01).

**Conclusion:**

In about one-third of acute ACL ruptures the medial collateral ligament complex is also injured. This might be associated with an increased knee laxity as well as anteromedial rotational instability. Also, this might be associated with an increased risk for failure of revision ACL reconstruction. In addition, we show risk factors and predictors that point to an injury of medial structures and facilitate their diagnosis. This should help physicians and surgeons to precisely diagnose and to assess its scope in order to initiate proper therapies. With this in mind, we would like to draw attention to a frequently occurring combination injury, the so-called “unlucky triad” (ACL, MCL, and lateral meniscus).

*Level of evidence* Level III Retrospective cohort study.

## Key points

- One-third of ACL ruptures are associated with injury to the medial complex
- There is significant correlation between an increased trauma severity affecting the posterolateral tibial plateau and the injury frequency / severity of the medial complex
- The “Unlucky triad” is a frequently observed combination injury of ACL rupture, MCL complex and lateral meniscus injury

## Background

The combined injury of the medial collateral ligament (MCL) complex and the anterior cruciate ligament (ACL) is the most common two ligament injury of the knee joint [1–3] and is linked to anteromedial rotatory instability (AMRI) [1]. Whereas there are common surgical and conservative concepts for the therapy of isolated ACL injuries, MCL therapy has received little attention, especially driven by the notion that non-surgical MCL therapy will lead to sufficient stability [4–7]. The MCL complex is the most important stabilizer against valgus stress and prevents antero- and posteromedial rotational instabilities in conjunction with the ACL in the form of load sharing [8–15]. The MCL complex exerts its complex function through its three main components: the superficial MCL (sMCL), the deep MCL (dMCL), and the posterior oblique ligament (POL). Especially with regard to anteromedial rotational instability (AMRI) these structures are of great importance [7, 16, 17] and excluding them from therapy planning can lead to an abnormally increased tibial rotational laxity [18]. This being especially problematic, given the recognized increased failure rates in primary and revision ACL reconstruction associated with MCL complex injuries, as well as their demonstrated connection to persistent medial instability [17, 19–21]. Yet, despite its frequency and recognition of its importance, a detailed evaluation of MCL complex injuries in the context of an ACL injury has so far received only little attention. Just recently, injuries of the dMCL have been described as often overlooked in supposedly isolated ACL tears [22]. In addition, the correlation between posterolateral bone bruising and higher pivoting energy during ACL injury is well established [23–26].

Posterolateral tibial plateau fractures reflect trauma severity and are associated with increased incidence of concomitant meniscal, capsuloligamentous injuries, and MCL complex injuries, specifically [24, 27]. However, there is currently a lack of detailed examination that differentiates between the sMCL, dMCL, and posteromedial capsule/POL structures, while also considering varying levels of trauma severity. Consequently, our primary objective was to provide a more thorough analysis of the injuries to the MCL complex associated with ACL ruptures, for a deeper understanding of their risk factors, concomitant pathologies and composition. The secondary objective was to correlate trauma severity affecting the posterolateral corner with the injury pattern. We hypothesize that trauma severity, defined by bone bruises or fractures to the posterolateral tibial plateau, correlates with the probability and extent of MCL complex injuries. To the best of our knowledge, this is the first scientific paper to provide a detailed analysis of MCL complex injuries in the case of ACL rupture in relation to trauma severity.

## Methods

### Patient selection

The inclusion criteria for this study were all patients who presented to our department at the University Medical Center Hamburg-Eppendorf with suspected rupture of the ACL and magnetic resonance imaging (MRI) between January 2017 and December 2018. Inclusion criteria also required patients to have available MRI scans, that were performed in the acute phase following trauma and were no older than 4 weeks. All of those who were diagnosed with a complete tear of the ACL, or tear of a prior ACL graft were included in the study. To investigate the presence of multiple ligament injuries, all additional ligament injuries were examined and included in the study. Exclusion criteria were type C fractures (AO/OTA) of the tibia or femur, chronic injury (> 6 months) of the ACL without reconstruction, and degenerative tears of the ACL. Age and sex were recorded for all patients.

### Imaging technique

Scans were performed on a 3.0 T MRI system (Ingenia, Philips, Best, The Netherlands) with a dedicated 16-channel knee coil (dStream, Philips, Best, The Netherlands) at maximum knee flexion of 5–10°. The MRI protocol was comprised of a T1/T2-FSE sequence in coronary plane and a fat suppressed PD-FSE sequence in transversal, sagittal and coronary plane.

### Image review

**Fig. 1:**
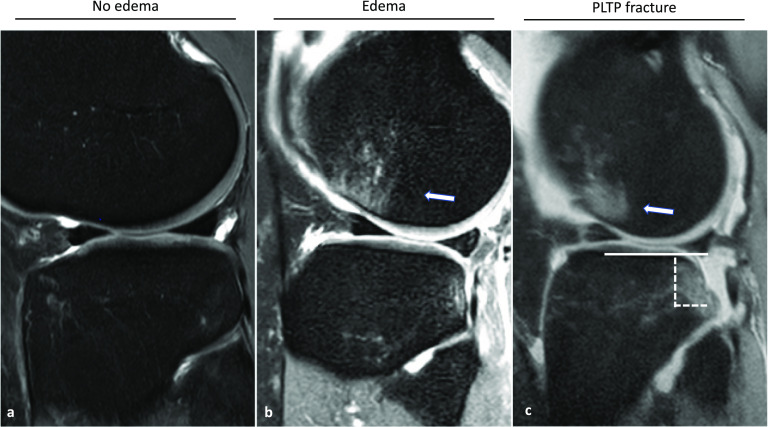
Exemplary representation of the Injury severity to the posterolateral tibial plateau. Sequential sagittal (**a**, **b**, **c**) fat-suppressed proton density-weighted magnetic imaging depicting the posterolateral aspect of the knee joint. In **a** there is no edema/no fracture caused in the event of an ACL rupture. **b** shows an edema of the PLTP with concomitant edema of the lateral femoral condyle (Indicated by the white arrow. In figure **c** we see a posterolateral fracture (2b according to Bernholt et al. [24]) with an indicated associated edema of the lateral femoral condyle (White arrow). The solid line indicates the physiological joint line. The dashed lines represent the impression fragment

MRI scans were independently reviewed by four observers, two radiologists (4- and 12-years’ experience in musculoskeletal MRI) and two trauma surgeons (2- and 12-years’ experience in musculoskeletal MRI). The posterolateral aspect of the tibial plateau was reviewed for the trauma severity in the course of the ACL rupture based on the configuration of the posterolateral tibial plateau. Three groups were distinguished: No edema/no fracture, edema and impaction fractures (Fig. 1).

All fractures, which could be diagnosed according to the classification system for posterolateral tibial plateau impaction fractures published by Bernholt et al. [24] were grouped together. The three groups are understood as a sign of an increasing pivotal trauma impact (No edema/no fracture > edema > impaction fractures) associated with acute ACL ruptures. A corresponding impression fracture or bone bruise of the lateral femur condyle was also noted.

**Fig. 2:**
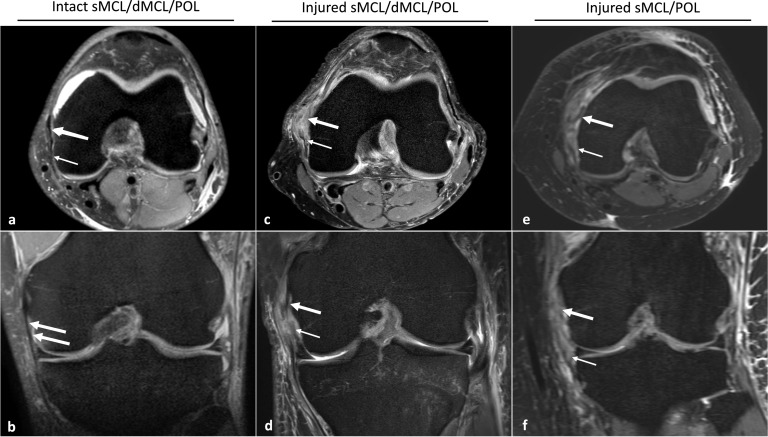
T2 weighted axial and coronal MRI sequences. Images (**a** and **b**) show an intact MCL complex. In the coronal **b** sequence the white arrows point to the intact POL and in the axial **a** sequence the large arrow points to an intact sMCL and the small arrow to an intact POL. Images (**c** and **d**) demonstrate a complete medial complex tear. In the axial **c** sequence the large white arrow points to the sMCL lesion and the small white arrow to the POL lesion. In the coronal sequence the large white arrow points to the sMCL lesion and the small white arrow to the dMCL lesion. In Images (**e** and **f**) one may observe coronal and axial sequences of a proximal rupture of the sMCL (large white arrows) and a POL lesion (small white arrows)

MRIs were scrutinized for accompanying medial soft tissue injury (Fig. 2). The relevant medial structures are the superficial and deep parts of the MCL, the medial meniscus (MM), and the POL. Injuries to the superficial and deep parts of the MCL were further classified as proximal, intermediate and distal or as proximal and distal injuries, respectively. Medial and lateral meniscus injuries were subdivided into anterior, intermediate, and posterior injuries, and ramp lesions. Each structure was rated as 0 (intact) or 1 (torn) [28]. Other relevant injuries were recorded (Table 1). These included medial tibial or femoral fractures or bone bruises; cartilage injury (only acute injury—old injuries or degenerative change was not reported); lateral collateral ligament (proximal, intermediate, and distal); the semitendinosus, biceps femoris, and popliteus muscles; and the popliteofibular ligament.

**Table 1:** Frequency of injuries of the medial collateral ligament complex in dependence of the trauma severity

Medial collateral ligament complex	Injury severity		
	No edema/No fracture	Edema	Fracture
MM total	9 (29%)	12 (16%)	7 (16%)
LM total	3 (10%	16 (26%)	5 (14%)
sMCL isolated	0 (0%)	0 (0%)	0 (0%)
dMCL isolated	3 (12%)	11 (21%)	6 (15%)
POL isolated	0 (0%)	0 (0%)	1 (3%)
sMCL/dMCL	1 (4%)	1 (2%)	1 (3%)
sMCL/POL	1 (4%)	0 (0%)	0 (0%)
sMCL/dMCL/POL	2 (8%)	2 (4%)	5 (13%)
dMCL/POL/MM	2 (7%)	2 (4%)	3 (7%)
dMCL/sMCL/POL/IM	0 (0%)	0 (0%)	0 (0%)
dMCL/sMCL/POL/AM	0 (0%)	1 (2%)	3 (8%)
without medial injury	19 (73%)	38 (72%)	23 (59%)

### Statistical analysis

Statistical analysis was performed using the statistical analysis software SAS 9.4 (SAS Institute Inc., Cary, NC, USA). Normal distribution was confirmed by the Shapiro–Wilk test and continuous variables were expressed as mean ± standard deviation. Binomial logistic regression analysis was performed to evaluate the risk factors for the presence of injuries to the MCL complex in the setting of ACL injury. Injury of the sMCL, dMCL or POL were the independent variables and injury trauma severity, sex, age, presence of other injuries of the MCL complex, a lateral or medial meniscus injury and fractures in the area of the lateral femoral condyle were used as dependent variables. The interrater reliability between the four readers was calculated via Cohen’s kappa value, using Chi^2^ test and Fisher exact test. The statistical level of significance was defined as *α* = 0.05 for all tests. In the implementation of the logistic regression the oversampling procedure was applied in some cases to improve the significance of imbalanced data [29–31]. Cases where oversampling was applied are marked with (by o.s.).

## Results

MRI examination was performed in 151 patients (male: *n* = 99; female: *n* = 52) with a mean age of 32.3 ± 11.8 (range, 15–71) years. 74 left and 77 right knees were included. 24 patients (15.9%) had no edema or fracture to the tibia or femur, 83 (54.9%) displayed edema of the posterolateral tibial plateau (PLTP) and 44 (29.1%) showed PLTP impaction fractures. An injury to the MCL complex was detected in 52 (34.4%) patients. The most commonly injured structure was the dMCL (*n* = 48, 92.3%). 20 (41.6%) patients had sustained an isolated injury to the dMCL. 25 (52%) cases were injured in combination with a rupture of the POL and 10 (20.8%) occurred in combination with an injury of the sMCL. The second most frequent MCL complex injury was a rupture of the POL (51.9%). Thereby isolated POL injuries were an exception (*n* = 1, 3.7%), while 25 (92.6%) POL lesions were associated with dMCL and nine (33.3%) with sMCL lesions. SMCL lesions were only detected in combination with other injuries to the MCL complex. Of the 11 ruptures of the sMCL, 9 occurred in combination with an injury of the dMCL and POL and two other sMCL lesions occurred with either associated dMCL or POL. An MCL complex associated injury to the lateral meniscus (LM) occurred in 34.6% of the patients and more often than to the medial meniscus (MM, 19.2%). Injuries to the MM occurred in in combination with injuries of the dMCL and the POL, but not in combination with the sMCL. Combined injuries of the dMCL and POL were accompanied by MM injury in 28%, 16% of which also presented an LM lesion. The majority of dMCL injuries (96%) occurred at the proximal portion (meniscofemoral ligament), whereas only two (4%) examinations demonstrated injury to the distal portion (meniscotibial ligament). sMCL injuries involved the proximal portion in seven (63.6%), the intraligamentous portion in one (9.1%), and the distal portion in two (18.2%) patients.

### Injuries of the medial collateral ligament complex depending on the trauma severity

**Fig. 3:**
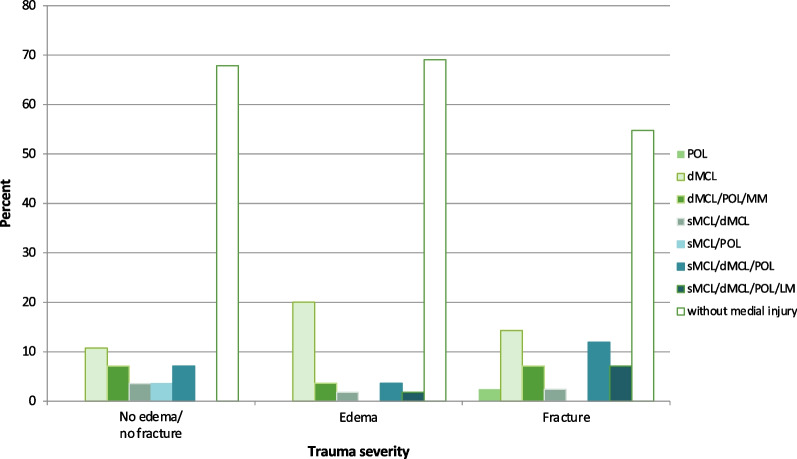
Occurrence of isolated and combined injuries of the medial collateral ligament complex depending on the trauma severity. From no edema/no fracture being the lowest, over edema to fractures of the posterolateral tibia plateau, representing the increased trauma severity. Most isolated dMCL injuries occurred at intermediate trauma severity and most combined injuries occurred at the highest trauma severity

**Fig. 4:**
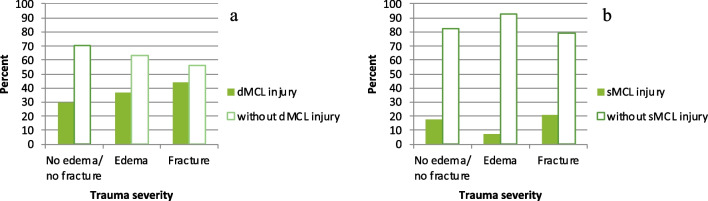
Represents the distribution of all dMCL (**a**) and sMCL (**b**) injuries in the case of an ACL rupture according to the trauma severity. Figures **a** and **b** both show a substantial increase in injuries in the event of a fracture, which is associated with increased trauma severity

While almost half of the patients with PLTP fractures showed no medial injury, the analysis of the individual MCL complex injury complexity revealed a significant association (Chi^2^ *p* < 0.0001) with trauma severity to the PLTP. Patients with PLTP fractures showed more complex medial injuries, like POL/dMCL/sMCL or dMCL/sMCL/LM), than patients with posterolateral bone bruises or no fracture (Fig. 3)*.* To determine risk factors for a MCL complex injury a logistic regression was performed. Patients with a dMCL injury were 13 times more likely to suffer a sMCL injury compared to patients with no sMCL injury (OR 13.033, 95% CI 1.3–133.3, *p* = 0.03). With a POL injury, the risk for dMCL injury is 22 times higher compared to patients with no POL injury (OR 22.263, 95% CI 5.9–84.4, *p* < 0.0001). Likewise, this association can be established for lesions of the POL and the sMCL. In addition, the likelihood of developing an sMCL injury increases sevenfold in patients with a POL injury (OR 6.629, 95% CI 1.3–35.1, *p* = 0.0261). Furthermore, trauma severity could be identified as a predictive factor for a dMCL lesion. The risk of exhibiting a dMCL injury was significantly raised in the case of presentation with a PLTP fracture (*p* < 0.02). Age, sex, and lateral femur fractures could not be identified as risk factors. Even though Figs. 3 and 4a, b strongly indicate the contrary, the logistic regression could furthermore not determine statistical prove for trauma severity as a risk factor for sMCL or POL injuries. Significant risk factors for acquiring an sMCL injury were age (*p* < 0.01) and injury to the LM (*p* < 0.01 (by o.s.)). Injuries to the MM were not risk factors.

The Cohen’s kappa value showed good interrater reliability of the analysis for isolated and combined injuries to the MCL complex (average cohen’s kappa = 0.70) as well as for the analysis of the injury to the PLTP (cohen’s kappa = 0.77) (Table 2).

**Table 2:** Cohen’s kappa value for interrater reliability between the four readers (two radiologists and two orthopedic surgeons)

	Cohen's kappa value
dMCL	0.68
POL	0.69
sMCL	0.81
dMCL/POL/sMCL	0.64
Bone edema/no bone edema or posterolateral impaction fractures	0.77

## Discussion

The main finding of this study was the varying extent of trauma severity to the posterolateral tibial plateau in correlation with an ACL rupture and its influence on the morphology of MCL complex injuries. In addition, we discovered that the sMCL never ruptured in isolation, but always in combination with either the dMCL or the POL or both together. The high number of injuries of the MCL complex (34.4%) associated with an ACL injury showed a clear correlation between increased trauma exposure and an increase in isolated and combined injuries. Thereby the most frequently injured structure was the dMCL (32.5%). Finally, we defined a frequently observed combined injury of ACL, MCL and LM as the “unlucky triad”.

The MCL complex consists of three main structures, namely the POL, dMCL, and sMCL, which together perform different functions in the stabilization of the knee joint against valgus and rotational stress [8, 10–15, 32, 33]. The sMCL primarily functions as a static stabilizer against valgus stress in almost all degrees of flexion (mainly in 20–30° of flexion), but less so in full extension [34–36]. Likewise, it acts as a stabilizer against sagittal stress [14] and external rotation (ER) [32]. The POL originates behind and slightly inferior to the proximal origin of the sMCL, running obliquely posteriorly and caudally to join the posteromedial capsule as well as inserting at the MM. It mainly stabilizes against valgus stress, more so in extension and tibial internal rotation (IR) [28, 34, 37, 38]. The dMCL has an oblique anatomy, running in a fan shape from its femoral attachment, posterior and distal to the sMCL femoral attachment, to its tibial attachment where it spreads out more broadly [32, 35, 39]. During tibial external rotation, the dMCL is thus tensioned, which underlines its function as a primary restraint to tibial ER in extension and as a secondary restraint to valgus rotation [14, 38, 40]. This explains its function as an ideal stabilizer against external rotation and anterior translation of the medial tibial plateau (AMRI) [35, 38, 39]. This being analogous to the oblique fibers of the anterolateral ligament (ALL) and its function as an important restraint against tibial rotation [41, 42], Athwal et al. [32] accordingly conclude, that the dMCL could be understood as the medial counterpart. Additionally, the dMCL participates in the stabilization against valgus stress. Due to its short length and deep position the probability of injury or rupture is higher than with the more superficial structures [14, 35, 43], which we were able to confirm in our work. In accordance with previous findings, the association between an ACL and MCL complex injury was common [1, 22, 24]. The incidence of 34.4% for an injury to the MCL complex is in line with the majority of previous reports, which present incidences of 22–44% of concomitant MCL injuries in the event of an ACL rupture [44–47]. Comparing our results with those of Willinger et al. [22], we have demonstrated fewer and no isolated sMCL injuries. In our understanding, this is due to our underlying radiological evaluation since we have only recorded complete ruptures. This is confirmed by the fact, that in their observation 100% of the MRI sMCL grade II injuries are accompanied by a dMCL injury. According to our observations 82% of sMCL ruptures occurred in combination with an injury to the dMCL and POL, thus demonstrating complete rupture of the MCL complex. This could explain why a clinical correlation with a MCL injury is usually mainly observed when the sMCL is also ruptured [3, 14, 48]. Namely, according to our data, this would be the equivalent to an injury of all medial structures of the MCL complex or at least of two out of three. Considering the different stabilizing roles and biomechanical functions of the individual structures of the medial knee, it can be concluded that an injury to the sMCL, associated with an ACL rupture, occurs in combination with other injuries to the MCL complex, which leads to increased valgus instability, both AMRI and posteromedial rotatory instability (PMRI). Furthermore Behrendt et al. [17] even describe a doubling of the degree of AMRI in the case of a combined injury of the sMCL and dMCL. According to our data the risk of a dMCL rupture is 13 times and that of a POL injury seven times increased in patients who have suffered a sMCL rupture. Our data have also shown that increasing patient age and additional LM injury are risk factors for the development of sMCL rupture. Interestingly, we were not only able to identify the classic injury combination of an unhappy triad, first described by O'donoghue D [49] as a combined injury to the ACL, MCL and MM. More often, however, a combination injury of the ACL, MCL and LM was observed. In relation to this, we describe fewer additional meniscus injuries (medial/lateral) compared to Willinger et al. [22]. We suspect that this is because we based our findings on purely radiological diagnostics and not on additional intraoperative findings. Nonetheless our observations confirm what has previously been pointed out in recent studies [26, 50] that besides the unhappy triad there is also another triad consisting of an ACL, MCL and LM injury, which we will henceforth refer to as the “unlucky triad”. In addition, it was found that this combination injury increases with increasing trauma severity (Fig. 3). Isolated injuries of the dMCL were observed in 45.1% of the cases. An injury that is often overlooked in clinical practice but may lead to persistent pain and increased ER [17, 51]. According to Narvani et al. [51] this prevented athletes from resuming professional sports and should therefore not be underestimated. Another main finding of this study was the varying extent of trauma to the PLTP and the associated varying expression of injuries to the MCL complex. We defined an increase in trauma severity from no edema/no fracture over edema and finally of fracture of the PLTP based on various descriptions of the current literature [23, 24]. With increase in trauma severity, there was a significant increase in dMCL injuries (*p* < 0.03). The POL and sMCL injuries also showed an increasing trend.

To the best of our knowledge, this work represents the first detailed analysis of the effect on the individual structures of the MCL complex (sMCL, dMCL, POL) associated with ACL rupture, with increasing trauma severity. As previously stated by Alm et al. [19] there is a 17-fold greater risk of revision anterior cruciate ligament reconstruction (ACLR) failure with persistent medial instability. According to their report the risk of failure of a primary ACLR in case of persistent medial instability increases by 13-fold. However, it is not specified which instability was caused by the injury of what structure and what influence in detail the injury of the individual components of the MCL complex have on the graft’s survival. Willinger et al. [22] describe that despite a previous radiological medial injury, there was no clinical correlate that would have indicated valgus laxity. Thus, on the one hand, medial injuries could easily be overlooked in the clinical examination and may also lead to instability in the course. On the other hand, the clinical examination possibilities to detect a rotational instability in the sense of an AMRI are limited, which might also increase the risk of developing an instability in the course. In our study we could show that firstly the dMCL is the most commonly injured structure within the MCL complex in association with an ACL rupture, with more frequent occurrence with increasing severity of trauma. Secondly, our findings suggest that sMCL injuries do not occur in isolation. Consequently, the presence of a sMCL injury on MRI indicates a heightened risk for medial instability. Recognizing the crucial role of these structures in stabilizing the knee and their impact on load distribution, we emphasize the necessity for increased attention to be given to their assessment and management in future clinical practice before operative treatment to determine the appropriate surgical approach.

## Limitations

This study has some limitations. This study included only radiographic information, which always offers the possibility for misinterpretation. In addition, the analysis of MRI imaging carries the risk of widely divergent assessments among the four independent investigators. However, our results demonstrated good interrater reliability between the four independent examiners, both for the diagnosis of trauma to the PLTP as well as capsuloligamentous structures. A further limitation of our study is the variability in the timing of MRI acquisitions following the trauma. Although the MRIs were obtained within a maximum timeframe of 4 weeks post-trauma, it is important to acknowledge that the size of bone bruise strongly correlates with the duration from injury and thus may vary within this time frame. Another limitation represents the absence of preoperative or postoperative clinical examinations so that we are unable to determine the potential effect of the individual injuries. The exact extent of the influence of these injuries on knee stability and also on the failure rate of ACL transplants will have to be the focus of further studies.

## Conclusion

In about one-third of acute ACL ruptures, there was an additional injury to the MCL complex. Isolated injuries of the sMCL were not observed. Risk factors for sMCL injury are increasing age and additional injury to the LM, POL and dMCL. In such a case, there were always combination injuries of the MCL complex. In this context, we present the “unlucky triad,” consisting of a combined injury to the ACL, MCL, and LM. The dMCL has been the most frequently violated structure in this context. A significant influence on the injury of the dMCL was an additional injury of the POL and the sMCL as well as an increasing trauma severity, which can be detected at the posterolateral tibial plateau.

## Data Availability

The datasets generated during and/or analyzed during the current study are available from the corresponding author on reasonable request.

## Abbreviations

ACL: Anterior cruciate ligament
ACLR: Anterior cruciate ligament reconstruction
ALL: Anterolateral ligament
ALRI: Anterolateral rotational instability
AMRI: Anteromedial rotational instability
dMCL: Deep MCL
ER: External rotation
FS: Fat-suppressed
FSE: Fast spin-echo
IR: Internal rotation
LM: Lateral meniscus
MCL: Medial collateral ligament
MM: Medial meniscus
MRI: Magnetic resonance imaging
PACS: Picture archiving and communication system
PD: Proton density
PLTP: Posterolateral tibial plateau
POL: Posterior oblique ligament
PMRI: Posteromedial rotatory instability
sMCL: Superficial MCL
TR: Repetition time
